# Temperature Modulates Plant Defense Responses through NB-LRR Proteins

**DOI:** 10.1371/journal.ppat.1000844

**Published:** 2010-04-01

**Authors:** Ying Zhu, Weiqiang Qian, Jian Hua

**Affiliations:** Department of Plant Biology, Cornell University, Ithaca, New York, United States of America; University of California Riverside, United States of America

## Abstract

An elevated growth temperature often inhibits plant defense responses and renders plants more susceptible to pathogens. However, the molecular mechanisms underlying this modulation are unknown. To genetically dissect this regulation, we isolated mutants that retain disease resistance at a higher growth temperature in Arabidopsis. One such heat-stable mutant results from a point mutation in *SNC1*, a NB-LRR encoding gene similar to disease resistance (*R*) genes. Similar mutations introduced into a tobacco *R* gene, *N,* confer defense responses at elevated temperature. Thus *R* genes or *R*-like genes involved in recognition of pathogen effectors are likely the causal temperature-sensitive component in defense responses. This is further supported by *snc1* intragenic suppressors that regained temperature sensitivity in defense responses. In addition, the SNC1 and N proteins had a reduction of nuclear accumulation at elevated temperature, which likely contributes to the inhibition of defense responses. These findings identify a plant temperature sensitive component in disease resistance and provide a potential means to generate plants adapting to a broader temperature range.

## Author Summary

It has been known that temperature modulates plant immune responses, but the molecular mechanisms underlying this modulation are unknown. Our study describes a novel finding that the NB-LRR type of R or R-like protein is the temperature-sensitive component of plant defense responses. R or R-like proteins have ‘receptor’ like functions involved in specific recognition of pathogens. Through genetic screens and targeted mutagenesis, we found that alterations in the *R*-like gene *SNC1* and the *R* gene *N* can change temperature sensitivity of defense responses. Further, an elevated temperature reduced the nuclear accumulation of SNC1, which likely contributes to the inhibition of disease resistance at high temperatures. Our study indicates that NB-LRR proteins mediate temperature sensitivity in plant immune responses.

## Introduction

Temperature is a major environmental factor that regulates plant growth and development as well as its interaction with other organisms [1]. Plants respond to small temperature changes and yet temperature signaling is largely unknown in plants [2]. Temperature is known to influence disease resistance to bacteria, fungi, virus, and insects; and different host-pathogen interactions respond differently to different temperature ranges [3]. A high temperature very often inhibits disease resistance or plant immunity [4], although low temperature also leads to reduced plant defense in some cases [5]. Despite the fact that temperature sensitivity poses a challenge to agriculture in the current global climate change scenario, the molecular basis for the high temperature inhibition of plant immunity is unknown.

Plant immunity occurs at multiple levels and can be largely divided into two branches. One is a general resistance responding to common features of pathogens named ‘microbial- or pathogen associated molecular patterns’ (MAMP or PAMP). The second immunity branch responds to pathogen virulent factors or effectors. This cultivar-specific resistance or ETI is induced upon a specific recognition of the pathogen race-specific avirulence (Avr) gene by disease resistance (*R*) gene of the host plant. This ‘gene-for-gene’ interaction leads to rapid and efficient defense responses including a form of programmed cell death named hypersensitive response (HR) to restrict the growth of pathogens. R proteins of the largest class have ‘nucleotide-binding’ (NB) and leucine-rich repeat (LRR) domains. The amino-termini of these proteins are either of the Toll and interleukin-1 receptor (TIR) type or the coiled-coiled (CC) type. Multiple layers of plant immunity reflect a co-evolution of host plants and pathogens.

Heat sensitivity of disease resistance has been observed in both basal defense responses and *R* gene-mediated defense response. For instance, Arabidopsis plants are more susceptible to virulent *Pseudomonas syringae pv. tomato* (*Pst*) DC3000 at 28°C than at 22°C [6]. Resistance to tobacco mosaic virus (TMV) conferred by the *N* gene is effective at 22°C, but is abolished at 30°C [7]. Resistance to root-knot nematodes conferred by the *Mi-1* gene in tomato is inactive above 28°C [8]. HR induced by the Arabidopsis *RPW8* gene against powdery mildew is suppressed by temperature above 30°C [9]. Arabidopsis resistance to avirulent *Pst* DC3000 strains with AvrRpt2, AvrRps4, or AvrRpm1 effectors exhibited at 22°C are inhibited at 28°C [6]. Resistance against fungal pathogen *Cladosporium fulvum* is conferred by *Cf4* and *Cf9* in tomato, and HR mediated by these two genes can be suppressed at 33°C [10]. A number of mutants with upregulated defense responses are also found to be temperature sensitive. The *bon1* mutant exhibits a dwarf phenotype at 22°C but not at 28°C due to a suppression of defense response mediated by *SNC1* at elevated temperature [11]. *SNC1* is a NB-LRR type of *R*-like gene closely related to the *R* genes *RPP4* and *RPP5* [12], and the gain-of-function mutant *snc1-1* exhibits a temperature-sensitive growth and defense phenotype [11]. Similarly, autoimmune response mediated by *R*-like genes in F1 hybrids between Arabidopsis accessions could be attenuated by a moderate increase in growth temperature [13].

The temperature effect on defense signaling is sometimes thought to be an indirect physiological change caused by global alterations in metabolism and membrane properties among others. However it is possible that a common mechanism for temperature sensitivity exists for different systems of disease resistance because many of them share similar signaling molecules and use similar signaling cascades. Some of the signaling components are themselves modulated by temperature. For instance, *EDS1* and *PAD4*, two regulators of both basal and *R*-mediated disease resistance, have a higher steady expression level at 22°C than at 28°C [11]. Salicylic acid (SA), a signal for systemic acquired resistance, is regulated by temperature [14],[15]. However, an initial attempt to alter temperature sensitivity by up-regulating *EDS1* and *PAD4* was not successful [6], and no systemic study had been carried out to investigate this temperature modulation of disease resistance at the molecular level.

Here we report a genetic screen for mutants with enhanced disease resistance at an elevated temperature. We show that the *R*-like gene *SNC1* and the *R* gene *N* are the temperature-sensitive components responsible for temperature sensitivity in defense responses they each induce. Alterations in R proteins are sufficient to change temperature sensitivity of plant immune response and confer defense responses at elevated temperature. Furthermore, a high temperature reduces nuclear localization of SNC1 and N proteins, which likely contributes to the repression of defense responses. Therefore, temperature sensitivity of R proteins is an important mechanism underlying temperature modulation of plant immunity.

## Results

### Isolation of a *int102* mutant that retains disease resistance at a high growth temperature

**Figure 1:**
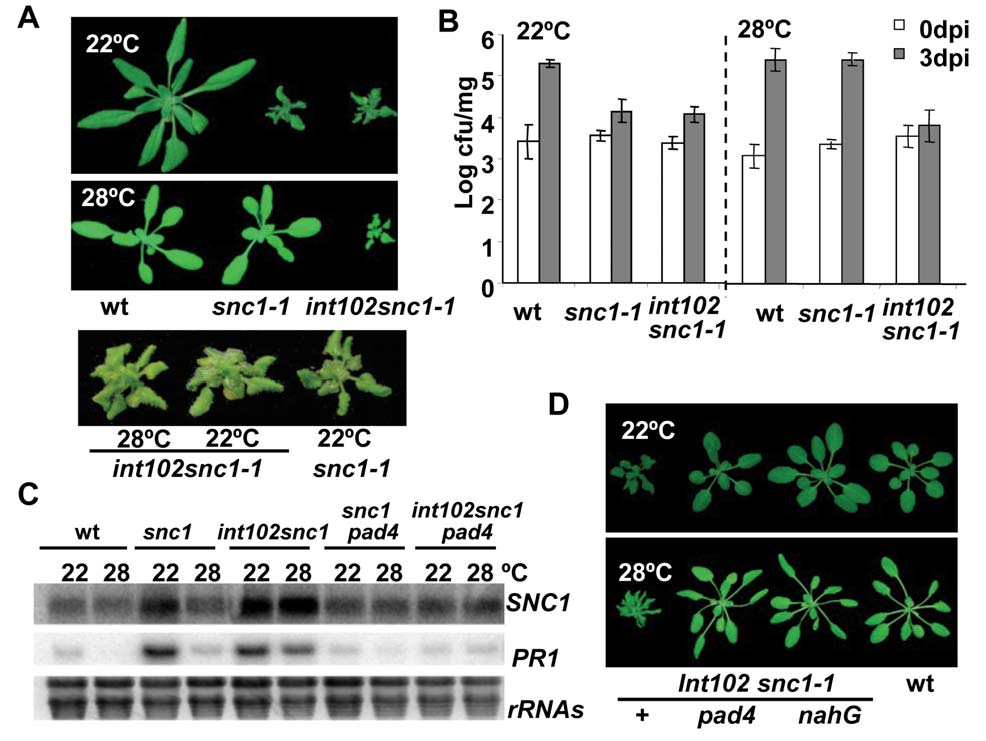
The *int102snc1-1* mutant has enhanced disease resistance at a high temperature. (A) The dwarf phenotype of *snc1-1* at 22°C is suppressed by a higher temperature of 28°C while the *int102 snc1-1* (*snc1-4*) mutant has the same dwarf phenotype at both 22°C and 28°C. Shown are plants of 4-week-old at 22°C and 3-week-old at 28°C. (B) The *int102* mutation enables *snc1-1* to retain resistance to a virulent pathogen at 28°C. Shown is the growth of *Pseudomonas syringae* pv tomato (*Pst*) DC3000 in the wild type, *snc1-1*, and *int102-1 snc1-1* (*snc1-4*) plants at 22°C and 28°C. The t-test shows significant difference in growth at 3 days post-inoculation (3 dpi) between the wild type and *int102 snc1-1* at both 22°C and 28°C (P = 0.007 and 0.001, respectively). It also shows significant difference at 3 dpi between the wild type and *snc1-1* at 22°C (P = 0.012) but not at 28°C (P = 0.415). Error bars represent standard deviations of three biological repeats. The experiments were repeated at least three times and similar results were obtained. (C) Expression of defense genes is upregulated in *int102-1 snc1-1* (*snc1-4*) at both 22°C and 28°C and this upregulation is dependent on *PAD4*. Shown are *PR1* and *SNC1* expressions in three-week-old plants analyzed by RNA blot. *rRNA*s were used as loading controls. (D) The dwarf phenotype of *int102-1 snc1-1* (*snc1-4*) is suppressed by *pad4* and *nahG*. Shown are wild type, *int102-1 snc1-1* (denoted as +), *int102-1 snc1-1 pad4*, and *int102-1 snc1-1 nahG* grown at 22°C and 28°C before bolting.

Wild-type Arabidopsis plants turn off defense responses in the absence of pathogens as these responses usually compromise plant growth and sometimes cause cell death. The Arabidopsis *snc1-1* mutant shows constitutive defense response and dwarf phenotype in a temperature-dependent manner, i.e., the mutant phenotypes are expressed at 22°C but are not at 28°C [11] (Fig. 1A). The growth regulation by temperature in *snc1-1* therefore serves as a model for investigating temperature modulation of defense responses. We carried out a screen for mutants that are defective in high-temperature inhibition of disease resistance in the *snc1-1* background by EMS mutagenesis. Mutants retaining a dwarf phenotype at 28°C together with *snc1-1* were isolated. One such mutant, *int (insensitive to temperature)102-1*, had an almost identical dwarf phenotype at both 22°C and 28°C; and it had a similar small size and a curly leaf shape as the *snc1-1* mutant grown at 22°C (Fig. 1A).

Further analysis revealed that the *int102-1 snc1-1* mutant retained enhanced defense responses at 28°C and was resistant to the bacterial pathogen *Pst* DC3000 at both 22°C and 28°C. At 22°C, the wild-type plants supported a 56-fold increase in bacterial growth after a 3-day inoculation, while *snc1-1* and *int102-1 snc1-1*supported only four- to five-fold increase in bacterial growth (Fig. 1B), indicating that *snc1-1* and *int102-1 snc1-1* exhibit enhanced resistance at a similar level at normal growth temperature. At 28°C, *snc1-1* was as susceptible as the wild type and supported 127-fold increase in bacterial growth. In contrast, there was little growth (two-fold increase) of bacteria on *int102-1 snc1-1* at 28°C (Fig. 1B). Therefore, the *int102-1 snc1-1* mutant indeed has elevated defense responses at both temperatures and the *int102-1* mutation confers a temperature-insensitive or heat-stable immune response.

We found that the elevated defense response in this mutant is mediated by salicylic acid (SA) and *PAD4*. The expressions of a defense response marker gene *PR1* [16] was higher in *int102-1 snc1-1* than in the wild type or *snc1-1* at 28°C (Fig. 1C), suggesting an activation of the SA pathway. The *nahG* transgene coding for SA degradation enzyme [17] was therefore introduced into *int102-1 snc1-1*, and this transgene indeed suppressed both the growth and defense gene expression phenotypes of *int102 snc1-1*. The *int102 snc1-1nahG* plants had wild-type morphology and no elevated *PR1* expression at either 22°C or 28°C (Fig. 1D and data not shown). Similarly, the *pad4* mutant defective in defense responses [18] suppressed the phenotypes of *int102-1 snc1-1* (Fig. 1C and D), further demonstrating an upregulation of defense responses in the *int102-1 snc1-1* mutant at 28°C.

### Mutation in the *R*-like gene *SNC1* is responsible for enhanced disease resistance at high temperatures

**Figure 2:**
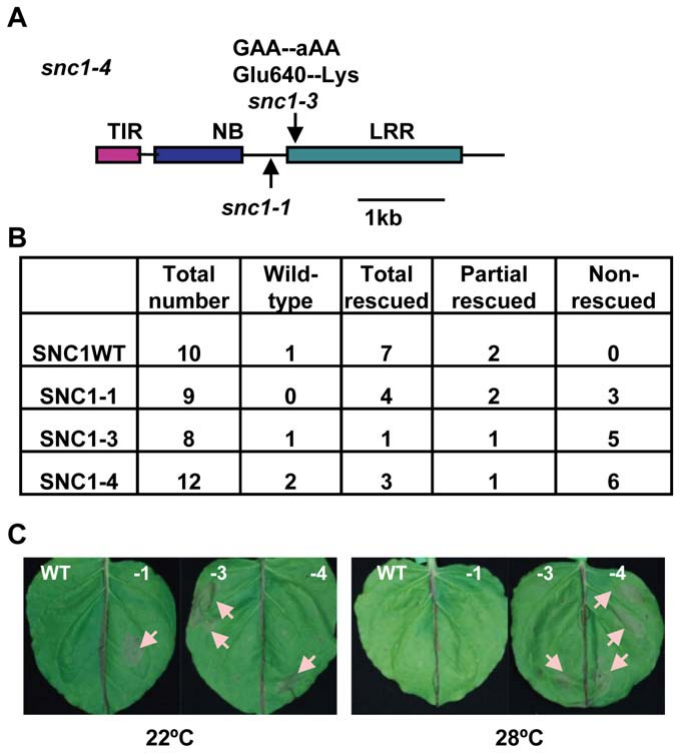
The *snc1-3* mutation confers enhanced defense responses at a high temperature. (A) Mutation sites of *snc1-1* and *snc1-3* in *int102 snc1-1* (*snc1-4*). Shown is the diagram of the coding sequence with the encoded protein domains of SNC1. The *snc1-3* mutation is in the LRR region. TIR, Toll Interleukin1 Receptor-like; NB, Nucleotide Binding; LRR, Leucine Rich Repeat. (B) Phenotypes of transgenic lines with *pSNC1::SNC1:GFP* constructs. Four *pSNC1::SNC1:GFP* constructs were analyzed: wild-type *SNC1WT*, *SNC1-1*, *SNC1-3*, and *SNC1-4*. Transgenic plants are categorized as wild-type like, dwarf at 22°C but rescued at 28°C (rescued), dwarf at 22°C but partially rescued at 28°C (partial rescued), and dwarf at 22°C and 28°C (non-rescued). Chi-square test shows that there is a significant difference between *SNC1-3* and *SNC1WT* (P = 0.021) and between *SNC1-4* and *SNC1WT* (P = 0.043), but not much difference between *SNC1-1* and *SNC1WT* (P = 0.189). (C) Hypersensitive Response (HR)-like cell death is induced by SNC1 with the *snc1-3* mutation at 28°C. Four *p35S::SNC1:GFP* constructs: *SNC1* wild-type (WT), *SNC1-1*, *SNC1-3* and *SNC1-4* (containing both *snc1-1* and *snc1-3* mutations), were transiently expressed in *Nicotiana benthamiana* (Nb) leaves via infiltration of a suspension of Agrobacterial cells harboring a specific construct to the whole leaf. At least six biological repeats were conducted for this set of strains and similar cell death-inducing activities were observed for each strain. Representative leaves at 3 days after infiltration are shown on the top panel and the lower panel shows the same leaves after clearing with ethanol. Infiltration sites (several per leaf) exhibited cell damage with a round shape. Cell death sites are indicated by arrows. Abbreviations: WT, SNC1WT:GFP; -1, SNC1-1:GFP; -3, SNC1-3:GFP; -4, SNC1-4:GFP.

We cloned the *INT102* gene based on its tight linkage to *SNC1* in a F2 population of *int102-1 snc1-1* (in the Col-0 accession) crossed to the wild-type Ws-2 accession. Analysis of 240 *int*-looking plants in this population revealed no recombination between *INT102* and *SNC1*, indicating a tight linkage of the two genes. Sequencing the *SNC1* gene in *int102-1 snc1-1* revealed a G to A point mutation causing a change of glutamic acid to lysine (named *snc1-3*) at amino acid residue 640 in the second LRR motif in the LRR domain of SNC1 (Fig. 2A, S1). The *int102-1 snc1-1* mutant is therefore named *snc1-4*, and it contains both the *snc1-1* and *snc1-3* mutations.

The *snc1-4* mutation is likely gain-of-function although it is recessive to the wild type and *snc1-1*. The same recessive property was observed for the gain-of-function mutation *snc1-1* [12]. This recessive nature is likely due to haploid deficiency rather than loss-of-function, and it is demonstrated that the mutant *SNC1-1* transgene induced *snc1-1* phenotype in transgenic plants [12]. To determine if the *snc1-3* mutation is the causal mutation of the *int* phenotype, we generated transgenic Arabidopsis lines containing different forms of *SNC1*. The SNC1 protein was tagged by the green fluorescent protein (GFP) at the carboxyl-terminus and expressed under the control of its native promoter (named as *pSNC1::SNC1:GFP*). Four forms of *SNC1* fusions were created: the wild-type (*SNC1WT*), *SNC1-1*, *SNC1-3*, and *SNC1-4*; and they were transformed into the wild-type Col-0 plants respectively. Primary transformants were grown first at 22°C for three weeks before being transferred to 28°C till seed setting and their growth phenotypes were scored both at 22°C and after two weeks at 28°C (Fig. 2B). Transgenic Arabidopsis plants with the same transgene exhibited varying phenotype, mostly likely due to varying expression levels of the transgene. Among the 10 *pSNC1::SNC1WT:GFP* lines generated, all but one exhibited morphological defects at 22°C indicating an activation of defense responses. This is consistent with the previous finding that the wild-type *SNC1* genomic fragment could induce autoactivation due to a higher *SNC1* expression level than the endogenous one [19]. Among these, seven lines showed a dwarf phenotype at 22°C but new tissues grown at 28°C did not exhibit visible growth defects and we termed these as rescued at 28°C. The rest two lines showed dwarf phenotype at 22°C but can be partially rescued at 28°C. Among the 9 lines of *pSNC1::SNC1-1:GFP*, all exhibited a dwarf phenotype. At 28°C, four lines were rescued, two lines were partially rescued, and 3 lines were not rescued. Among the 8 lines of *pSNC1::SNC1-3:GFP*, one did not exhibit a morphological defect, one had a 28°C rescued dwarf phenotype, one had a partial 28°C rescued phenotype, and 5 had a non-rescued phenotype. Among the 12 lines with *pSNC1::SNC1-4:GFP*, one did not exhibit a growth defect, three had a 28°C rescued dwarf phenotype, one had a partial rescued and 6 had a 28°C non-rescued phenotype. In sum, the *SNC1-3* and *SNC1-4* constructs induced a significantly higher percentage of transgenic lines with a dwarf phenotype at both 22°C and 28°C, indicating that the *snc1-3* mutation is the causal mutation of *int102* and that the *snc1-3* mutation alone (without *snc1-1*) is sufficient to induce defense responses at a higher temperature. Therefore, temperature sensitivity of disease resistance induced by *SNC1* is controlled by the *R*-like gene itself rather than by other regulatory components and that a mutation in the *R*-like gene is sufficient to confer disease resistance at a high temperature.

### The *SNC1-4* mutant gene induces HR-like cell death at elevated temperature in *Nicotiana benthamiana*

We explored the possibility of using a transient assay to analyze the SNC1 activity and its protein localization, as we could not detect SNC1:GFP signals by microscope in transgenic Arabidopsis. Because many *R* genes induce HR-like cell death in *N. benthamiana* (Nb) when co-expressed with their elicitors and some activated forms of *R* genes induced cell death in the absence of their elicitors, we tested cell death-inducing activities of different forms of SNC1 expressed under a strong 35S promoter by *Agrobacterium*-mediated infiltration (agro-infiltration) in Nb. For agro-infiltration experiments of more than six replicas, we saw a correlation of cell death-inducing activities in Nb with defense response (dwarf)-inducing activities in Arabidopsis for different forms of *SNC1* genes (Fig. 2C). No visible cell death was observed for *p35S::SNC1WT:GFP* at 22°C or 28°C. On the other hand, the *p35S::SNC1-1:GFP* fusion induced visible cell death at 22°C but not at 28°C. In fully infiltrated leaves, small areas (more than 2 mm in diameter) underwent cell death manifested by collapsed leave cells. In contrast, both *p35S::SNC1-3:GFP* and *p35S::SNC1-4:GFP* induced visible cell death at both temperatures. These results further support the conclusion that *snc1-3* mutation is the causal mutation for heat-stable resistance.

### The *rit1* mutation suppresses defense responses in *snc1-4* only at elevated temperature

**Figure 3:**
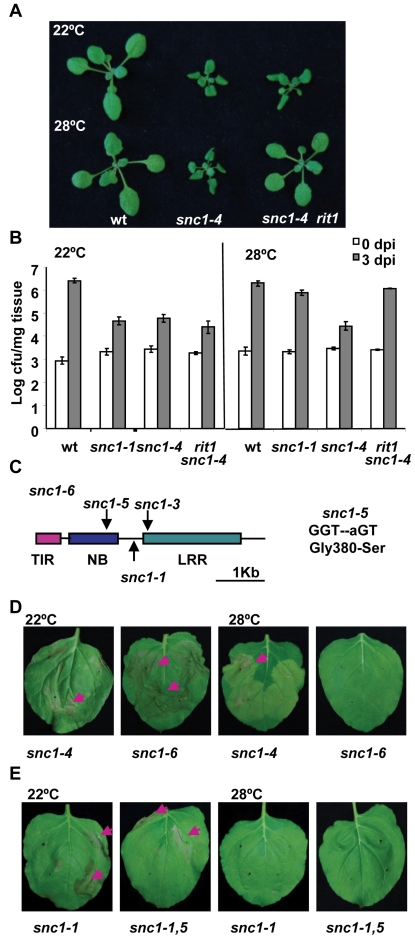
The *snc1-5* mutation reverts the heat-stable resistance of *snc1-4*. (A) The *rit1* mutant regained a temperature-sensitive growth phenotype. The *snc1-4* mutant had dwarf phenotype at both 22°C and 28°C compared to the wild-type Col while *rit1 snc1-4* was wild-type looking at 28°C but dwarf at 22°C. (B) The *rit1* mutant has enhanced disease resistance at 22°C but not at 28°C. Shown is the growth of *Pst* DC3000 in the wild type, *snc1-1*, *snc1-4* and *snc1-4 rit1* plants at 22°C and 28°C. The *snc1-4* mutant is more resistant than the wild type at both 22°C and 28°C while the *snc1-4 rit1* mutant is more susceptible than *snc1-4* at 28°C but as resistant to *snc1-4* at 22°C. The t-test on growth at 3 dpi at 22°C shows that there is a significant difference between the wild type and the other three mutants: *snc1-1*, *snc1-4*, and *snc1-4 rit1* (P = 0.0007, 0.0001, and 0.0012, respectively) and that there is no significant difference between *snc1-4* and *snc1-4 rit1* (P = 0.13). The t-test on growth at 3 dpi at 28°C shows that there is a significant difference between *snc1-4* and the wild type or *snc1-1* (P = 0.003, 0.001, respectively) and between *snc1-4* and *snc1-4 rit1* (P = 0.004) but no significant difference between *snc1-1* and *snc1-4 rit1* (P = 0.126). Error bars represent standard deviations of three biological repeats. The experiments were repeated two times and similar results were obtained. (C) Diagram of mutation sites in *snc1-6*. The *snc1-5* mutation is in the NB-ARC domain. (D) Activity assay of the *SNC1-6* mutant gene in Nb. Shown are Nb leaves three days after agro-infiltration with *SNC1-4:GFP* and *SNC1-6:GFP*. While *SNC1-4* induces cell death at 22°C and 28°C, while *SNC1-6* induces cell death only at 22°C but not 28°C. (E) Effect of *snc1-5* mutation on *SNC1-1* activity. Shown are Nb leaves three days after agro-infiltration with *SNC1-1:GFP* and *SNC1-1,5:GFP*. Both *SNC1* forms induced cell death at 22°C but not 28°C.

To further investigate the mechanism underlying temperature sensitivity of defense responses, we carried out a suppressor screen in the heat-stable *snc1-4* mutant background. Mutants that regained high-temperature inhibition of defense responses were isolated and named *rit* (*revertant of int*). One such mutant *rit1 snc1-4* had a *snc1-1* like phenotype: dwarf at 22°C but wild-type like at 28°C, suggesting a regaining of temperature sensitivity (Fig. 3A). Correlated with its growth defect, the *rit1 snc1-4* mutant has an enhanced disease resistance to virulent pathogen *Pst* DC3000 at 22°C and this elevated defense is suppressed at 28°C (Fig. 3B). At 22°C, *Pst* DC3000 had a similar growth reduction in *rit1 snc1-4* as in *snc1-1* and *snc1-4* compared to the wild-type Col (p = 0.24, 0.11 respectively). At 28°C, *snc1-4* exhibited an inhibition of bacterial growth to a similar extent as at 22°C (p = 0.065). In contrast, *rit1 snc1-4* lost the inhibition of bacterial growth at 28°C conferred by *snc1-4* (p = 0.0001) and supported bacterial growth to a similar extent as *snc1-1* (p = 0.064). Therefore *rit1* indeed reverses the heat-stable resistance phenotype to the heat-sensitive phenotype.

### The *rit1* mutant has a missense mutation in *SNC1*

We found that *rit1* is an intragenic suppressor of *snc1-4*. There is no phenotypic segregation in the F2 progenies of a cross of *rit1 snc1-4* with wild-type Col-0 or L*er* grown at 28°C indicating that the *rit1* mutation is very closely linked to the *SNC1* gene. Sequencing the entire *SNC1* genomic fragment in the *rit1 snc1-4* mutant identified a G to A point mutation resulting in a serine substitution of glycine at amino acid residue 380 (Fig. 3C, S1). We named this G380S mutation *snc1-5* and this new allele with *snc1-1*, *snc1-3*, and *snc1-5* mutations as *snc1-6* (Fig. 3C). This glycine residue resides immediately after the putative GxP or GLPL motif in the NB-ARC domain. This motif was previously identified as important for nucleotide binding and mutations in residues close to the motif might compromise activation of NB-LRR proteins [20].

A second intragenic suppressor named *rit4* was identified from the same *rit* screen. This mutant was independent of *rit1* as it was isolated from a different mutagenesis pool and had an additional phenotype unrelated to defense. Interestingly, we found the same G to A alteration resulting in a G380S mutation as in *rit4*. That two independent but identical mutations result in the same *rit* phenotype confirms that *snc1-5* is indeed the mutation responsible for reverting the temperature insensitivity of *snc1-4*.

This conclusion is further supported by the SNC1-6 activity in the Nb transient expression system. The *snc1-5* mutation was introduced into the *p35S::SNC1-4:GFP* construct to create *p35S::SNC1-6:GFP*. While *SNC1-4* induced cell death in Nb at both 22°C and 28°C, *SNC1-6* induced cell death only at 22°C but not at 28°C (Fig. 3D). Thus *snc1-5* mutation appears to be a suppressor of the heat-stable SNC1-4 activity specifically at 28°C and it does not significantly suppress SNC1-4 activity at 22°C. This notion is further supported by the failure of inhibiting the SNC1-1 22°C activity by the *snc1-5* mutation at 22°C. When the *snc1-5* mutation was introduced into *SNC1-1*, the *p35S::SNC1-1,5:GFP* was able to induce cell death at 22°C but not at 28°C similarly to *p35S::SNC1-1:GFP* (Fig. 3E).

With the identification of different forms of *SNC1* conferring defense responses of different temperature sensitivity, we conclude that the NB-LRR gene *SNC1* is the temperature-sensitive component causing temperature sensitivity of the whole defense responses it induces. An elevated temperature inhibits plant immunity probably through the very early component of the signaling pathway that is the *NB-LRR* genes.

### Temperature affects subcellular accumulation of the SNC1 proteins

We further investigated the molecular basis underlying the temperature sensitivity of *SNC1*. The *SNC1* transcript level was higher in *snc1-1* than in the wild type at 22°C but not at 28°C, while it was higher in *snc1-4* at both temperatures (Fig. 1C). Consistent with previous findings [11],[19], regulation of *SNC1* at the transcript level is largely due to a feedback amplification through *PAD4*, as the *snc1-4 pad4* double mutant had the same amount of *SNC1* transcript as *snc1-1 pad4* (Fig. 1C). Therefore, upregulation of the *SNC1* transcript by the *snc1-3* mutation at a high temperature is unlikely the primary cause of the heat-stable resistance.

Because an extremely high temperature of 37°C was shown to greatly inhibit the accumulation of the NB-LRR type of R protein MLA [21], we investigated whether temperature sensitivity is due to differential accumulation of SNC1 proteins at 22°C and 28°C. In transgenic Arabidopsis with *pSNC1::SNC1:GFP*, we were able to detect weak GFP signals by Western although no GFP signals could be visualized by microscopy. Three independent lines with each of the four wild-type and mutant *SNC1* transgenes were analyzed by protein blots for GFP expression at 22°C and 28°C (Fig. S2, Text S1). No dramatic differences in expression level were observed among different SNC1 versions or between 28°C rescued and 28°C non-rescued lines. There is a slight reduction of SNC1:GFP at 28°C compared to at 22°C in the 28°C rescued lines but not much in the non-rescued lines. Whether this reflects a biologically significant reduction or a dilution of signals by non-transgenic plants from the segregating population is yet to be determined.

**Figure 4:**
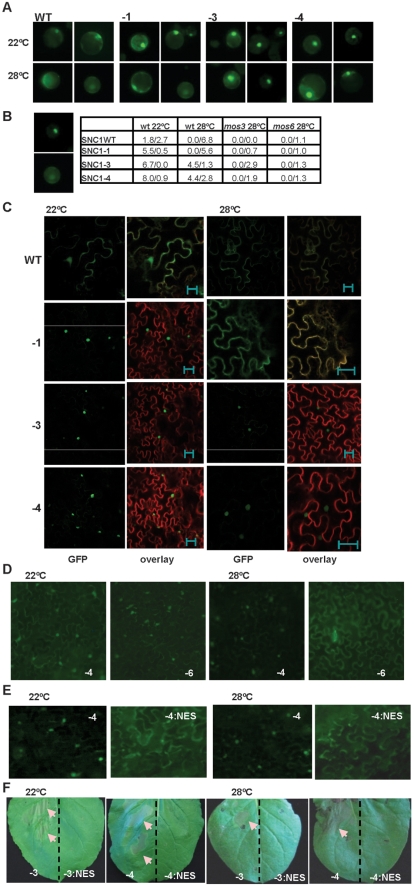
Nuclear localization of the *SNC1-3* protein is likely critical for disease resistance at elevated temperature. (A) Expression of SNC1:GFP in Arabidopsis protoplasts. Protoplasts isolated from the wild-type Col-0 plants were transformed with the same set of p35S::SNC1:GFP (WT, -1, -3 and -4) as in Fig. 2C, and the protoplasts were incubated at 22°C and 28°C for 12 hours before the signals were analyzed with a ZEISS AXIO scope. Two representative pictures of each transformation are shown. (B) Quantification of SNC1:GFP localization in the wild type (wt) and the mutant (*mos3* and *mos6*) protoplasts. Shown on the left are representative images of ‘nucleus only’ signal (top) and ‘ubiquitous signal’ (bottom). Shown on the right are percentages of cells with the nucleus-only expression versus the percentage of cells with ubiquitous localization. Data for cells of ‘no nucleus’ category are not shown. (C) Subcellular localization of the SNC1:GFP proteins in Nb at 22°C and 28°C. Infiltrated leaves as described in Fig. 2C were analyzed by confocal microscopy one day before the onset of cell death. Shown are the GFP signals (green), and overlay of signals from GFP and the membrane stain FM4-64 (red). Images were taken with Leica TCS SP5 confocal microscope. Nuclear localization was observed for SNC1-1, SNC1-3, and SNC1-4 at 22°C as well as SNC1-3 and SNC1-4 at 28°C. The bar represents 20 µm. (D) Subcellular localization of SNC1-6:GFP in Nb at 22°C and 28°C. The *snc1-5* mutation abolishes nuclear localization of SNC1-4 at 28°C but not at 22°C. (E) A nucleus export signal (NES) reduces the nuclear localization of SNC1-4. *SNC1-4:GFP* and *SNC1-4:NES:GFP* were agro-infiltrated in Nb. Shown are GFP signals at 22°C and 28°C taken with a ZEISS AXIO 2 plus scope. SNC1-4:NES:GFP had a weaker signal than SNC1-4:GFP, and a longer exposure time was used for its imaging. (F) The NES abolishes the activity of SNC1-3 and SNC1-4. *SNC1-3:GFP*, *SNC1-3:NES:GFP*, *SNC1-4:GFP* and *SNC1-4:NES:GFP* were agro-infiltrated in Nb and their cell death inducing activities were assayed at 22°C and 28°C. The left half of each leaf was infiltrated with the SNC1:GFP fusions, and the right half of the leaf was infiltrated with the corresponding SNC1:NES:GFP fusions. Shown are leaves at 3 days after infiltration. cell death indicated by arrows was only observed with fusion proteins without NES. Abbreviations: WT, SNC1WT:GFP; -1, SNC1-1:GFP; -3, SNC1-3:GFP; -4, SNC1-4:GFP; -3:NES, SNC1-3:NES:GFP; -4:NES, SNC1-4:NES:GFP; -6, SNC1-6:GFP.

A significant correlation was observed between the amount of nuclear accumulation of the SNC1 protein and its activity. Because we could not detect GFP signals in transgenic plants, we transiently expressed different forms of *p35S::SNC1:GFP* in Arabidopsis protoplasts. Despite a low expression efficiency compared to *p35S::GFP*, three expression patterns were observed for the SNC1:GFP proteins: nucleus-only, ubiquitous (cytosol, plasma membrane, and nucleus), and no nucleus (cytosol or cytosol and plasma membrane) (Fig. 4A, B). As it was difficult to distinguish no nucleus signal from background, we scored the expression patterns by the first two categories from 200 to 300 protoplasts for each transformation. For protoplasts transformed with SNC1WT at 22°C, 1.8% showed nucleus-only signal and 2.7% showed ubiquitous signal. In contrast, SNC1-1, SNC1-3, and SNC1-4 exhibited nucleus-only signal but no ubiquitous signal (Fig. 4B). At 28°C, neither SNC1WT nor SNC1-1 exhibited the nucleus-only signal, while the majority of SNC1-3 and SNC1-4 cells exhibited the nucleus-only signal (Fig. 4B). Therefore, there is a general correlation of high SNC1 activity with more nuclear signal. At 22°C, all three mutant forms have a higher nuclear accumulation than the wild-type form. At 28°C, nuclear accumulation of SNC1WT and SNC1-1 but not that of SNC1-3 or SNC1-4 was reduced. Thus an elevated temperature reduces the nuclear accumulation of the SNC1 protein and certain mutations such as *snc1-3* could resist this inhibition and induce defense at high temperatures.

### Nuclear accumulation of SNC1 at elevated temperature might be critical for its activity

A similar inhibition of nuclear accumulation by temperature was also observed for SNC1 expressed in Nb (Fig. 4C). A very weak GFP signal was detected for the SNC1WT:GFP fusion protein and the signal was mostly in the cytosol and plasma membrane. On the other hand, mutant fusions proteins of SNC1-1, SNC1-3 and SNC1-4 had higher fluorescent signals and the signals were mainly localized to the nucleus at 22°C. At 28°C, SNC1-1:GFP was found in the cytosol and plasma membrane and no nucleus localization was observed. In contrast, SNC1-3:GFP and SNC1-4:GFP were both mostly accumulated in the nucleus with SNC1-4 with a stronger signal. We further tested the effect of *snc1-5* mutation on the localization of SNC1-4 protein. Correlated with the cell death inducing activity at 22°C but not 28°C, SNC1-6:GFP is localized in the nucleus at 22°C but not 28°C when expressed in Nb (Fig. 4F). Thus, an elevated temperature could reduce the nuclear accumulation of heat-sensitive SNC1-1 and SNC1-6 but not the heat-stable SNC1-3 and SNC1-4, indicating that nuclear localization of SNC1 might be critical for its activity.

The nuclear accumulation of SNC1 appears to be required for the enhanced defense responses at elevated temperatures. A nucleus export signal (NES) [22] was added to the SNC1-4:GFP fusion, and it abolished the nuclear localization of SNC1-4:GFP at 28°C and also resulted in a weaker expression of the protein (Fig. 4E). The resulting SNC1-4:NES:GFP could no longer induce cell death at either 22°C or 28°C in contrast to SNC1-4:GFP (Fig. 4F). Similar activity suppression by NES was also observed for the SNC1-3:GFP fusion (Fig. 4F). Thus, nuclear localization at high temperatures is likely critical for the mutant SNC1 proteins to induce heat-stable defense responses. It remains however to be determined if the total amount of SNC1-4:NES:GFP expression is reduced compared to SNC1-4:GFP, and if so whether the SNC1-4 protein becomes less stable outside the nucleus.

### Nuclear accumulation of the SNC1 protein is an early event in activating defense responses at elevated temperature

**Figure 5:**
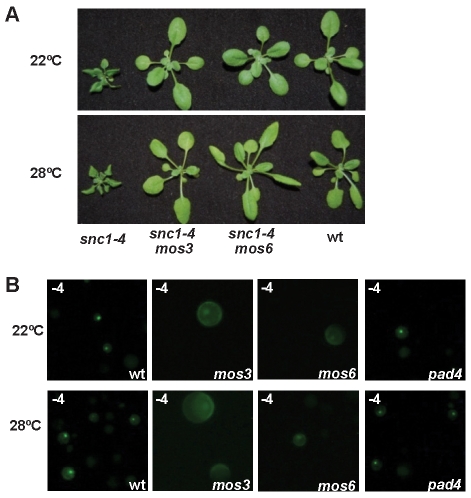
Effects of *mos3*, *mos6*, and *pad4* on the activity and localization of SNC1-4. (A) The dwarf phenotype of *snc1-4* is suppressed by *mos3* and *mos6*. Shown are 3-week-old seedlings of the wild type, *snc1-4 mos3*, *snc1-4 mos6*, and *snc1-4* at 22°C and 28°C. (B) Subcellular localization of the SNC1-4:GFP protein at 22°C and 28°C in wild-type (wt), *mos3*, *mos6*, and *pad4* protoplasts. Images were taken with ZEISS AXIO scope with the same setting.

To assess the relative position of nuclear accumulation of SNC1 in the signaling event of disease resistance at high temperature, we analyzed SNC1-4 localization in a few mutants that suppress the *snc1-4* mutant phenotype. In addition to *PAD4* and SA, we found that *MOS3* and *MOS6*, two genes required for *snc1-1* activity [23],[24], are also required for disease resistance in *snc1-4* at high temperature. Both the *snc1-4 mos3* and the *snc1-4 mos6* double mutants exhibited a largely wild-type growth phenotype at 22°C and 28°C (Fig. 5A).

We found that the nuclear accumulation of SNC1-3 and SNC1-4 at 28°C was inhibited by the *mos3* and *mos6* mutations. No protoplasts showed nucleus-only signal in *mos3* or *mos6* for SNC1-4 (Fig. 5B, 4B). In contrast, the loss-of-function mutation of *PAD4* did not alter the nuclear accumulation of the *SNC1* mutant proteins (Fig. 5B) although it suppressed the *snc1-4* mutant phenotype (Fig. 1C, D). These data suggest that *MOS3* and *MOS6* mediate *SNC1*-induced defense responses via an early event influencing the localization of R-like protein. *MOS3* encodes a putative nucleoporin Nup96 which could be responsible for RNA transport through nuclear envelope. It is yet to be determined how it influences the SNC1 localization. *MOS6* encodes a putative importin α3, which may affect R protein shuttling directly. That *pad4* affects disease resistance but not SNC1 localization in protoplasts further indicates that nuclear localization of the SNC1 protein at high temperature is a critical early event in heat-stable defense responses.

### Mutations in the *R* gene *N* confer defense responses at elevated temperature

**Figure 6:**
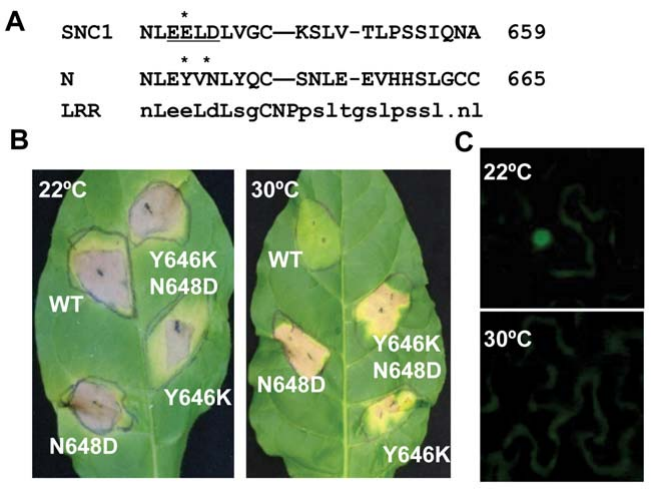
Mutant *N* genes induce defense responses at a high temperature. (A) Alignment of the LRR segment of the SNC1 and N proteins containing the *snc1-3* mutation site. Consensus sequences of this LRR segment are shown at the bottom. The potential sumoylation motif when with an E648K mutation is underlined. Residues mutated in this study are indicated by *. (B) Shown are cell death induced by the WT and three mutant *N* genes at 22°C and 30°C in *Nicotiana tobaccum* (Nt). The WT and mutant *N* genes were agro-infiltrated together with its elicitor p50 in Nt. The WT *N* gene induced cell death at 22°C but not 30°C, while the *N* mutant genes with Y646K, N648D, or Y646KN648D induced cell death at both temperatures. (C) Subcellular localization of the N protein in Nb at 22°C and 30°C when co-expressed with p50. The N-citrine chimeric gene was agro-infiltrated in Nb together with its elicitor p50, and the citrine signal was monitored up to three days after infiltration. Nuclear localization of the N-citrine protein was observed at 22°C but not at 30°C.

To determine if our observation of temperature-sensitive induction of defense responses by the Arabidopsis *R*-like gene *SNC1* is a general phenomenon for NB-LRR type of *R* genes, we analyzed defense response induced by the *R* gene *N*, which confers resistance to tobacco mosaic virus (TMV) only at temperatures below 28°C [7],[25]. A mutation of Y646K corresponding to *snc1-3* (E640K) was introduced into a genomic fragment of *N* gene described previously [26] (Fig. 6A). Because the EK640LD motif in SNC1-3 forms a potential sumoylation site, we also made a mutation in the *N* gene to introduce a N648D change corresponding to D642 of SNC1 so that the double mutant Y646K N648D could potentially provide a sumoylation site in the N protein as in the Arabidopsis SNC1-3 protein (Fig. 6A). When co-expressed with its elicitor p50 in *Nicotiana tobaccum* (Nt) by agro-infiltration, the wild-type *N* gene triggered HR-like cell death at 22°C but not at 30°C (Fig. 4B). In contrast, mutant *N* genes containing Y646K, or Y646KN648D mutations, when co-expressed with p50, induced cell death in Nt at both 22°C and 30°C (Fig. 6B). To our surprise, the N648D mutant *N* gene also induced cell death at 30°C (Fig. 6B). These mutations do not appear to confer constitutive auto-activities because they did not cause cell death in the absence of p50. Thus, the *N* gene is responsible for the temperature sensitivity of HR associated with TMV resistance, indicating that other NB-LRR type of *R* genes might also function as temperature-sensitive components in defense responses and that temperature sensitivity can be altered by specific mutations in the R proteins to confer heat-stable disease resistance.

We determined if temperature sensitivity of the *N*-mediated defense response is correlated with the N protein localization similarly to that of the SNC1 protein. A fusion protein of N and citrine was shown previously to be localized to the nucleus when expressed together with p50 in *N. benthamiana* [27]. We found that in contrast to nucleus localization of N:citrine at 22°C when co-expressed with p50 (Fig. 6C), no signal could be detected in the nucleus when infiltrated plants were incubated at 30°C (Fig. 6C). This indicates that nuclear localization of activated wild-type R protein(s) is subject to temperature modulation, similar to active form of the mutant R protein SNC1-1.

## Discussion

Despite the fact that temperature regulates many different growth and developmental processes, the temperature-sensitive components that control this sensitivity are largely unknown in plants. Temperature sensitivity in plant disease resistance is a phenomenon reported as early as 1969 and observed in various plant-pathogen interactions. Through a genetic screen for Arabidopsis heat-stable mutants that would retain defense responses normally inhibited at elevated temperatures, we identified the NB-LRR type of *R*-like gene *SNC1* as a key component responsible for temperature sensitivity. A point mutation in the *SNC1* gene is sufficient to induce defense responses at an elevated temperature (Fig. 1). Through a second genetic screen, we identified a *SNC1* mutation that appears to inhibit *SNC1* activity specifically at high temperature (Fig. 5). This finding reinforces the notion that the NB-LRR encoding gene *SNC1* is temperature sensitive. Furthermore, a mutation similar to the heat-stable mutation identified in *SNC1* was created in the *N* gene, and the mutant *N* gene was capable of inducing HR at a higher temperature (Fig. 6), indicating that the *R* gene *N* is responsible for temperature sensitivity of *N*-mediated defense responses. Thus we uncovered a mechanism for high temperature inhibition of plant immune responses. It is very likely that the NB-LRR type of *R* genes rather than other signaling components are responsible for temperature sensitivity in many other *R*-mediated disease resistance. This mechanism may also account for temperature sensitivity in some lesion mimic mutants and hybrid necrosis. In those mutants or hybrids, upregulated defense responses and lesions could have arisen at least in part from *R* gene activation similarly to upregulation of the *R*-like genes in the *bon1* or *bon1bon3* mutants [28],[29]. Inhibition of *R* or *R*-like activity by a higher growth temperature could suppress cell death and defense responses induced by those *R* or *R*-like genes.

It is not obvious if there is any selective advantage to have a temperature-sensitive immune system. Structural constraints may have prevented the evolution of heat-stable *R* genes that are also properly regulated. *R* genes with heat-stable activity could be associated with fitness cost, which may not manifest in the laboratory. Nevertheless, heat-stable resistance does occur in nature. For instance, the tomato *Mi-9* gene confers a heat-stable resistance to root-knot nematodes. Though the gene is not yet cloned, it is shown to be a homolog of the heat sensitive *Mi-1* gene [30]. It will be interesting to see if any of them arose from changes in the *R* genes similar to *snc1-3*.

**Figure 7:**
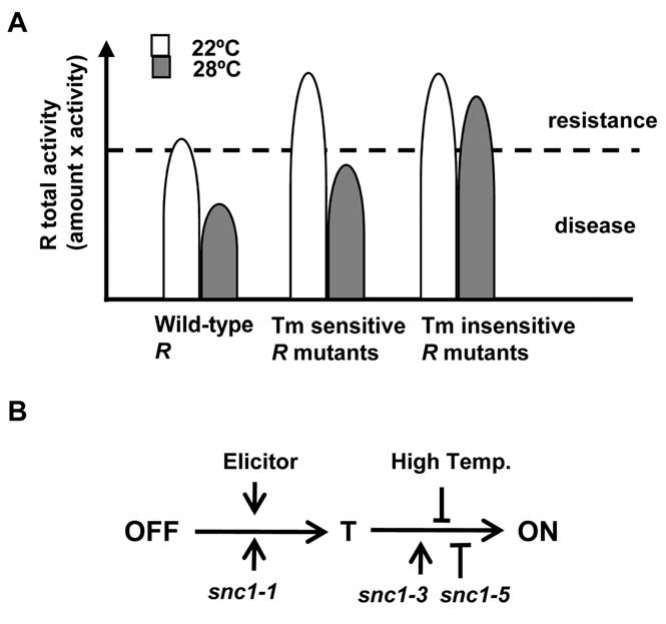
Model of temperature sensitivity of plant defense responses. (A) Model of temperature modulation of defense responses through the total R activity. The threshold of disease resistance is indicated by the dotted line. The activity of R is inhibited by an elevated temperature. Some forms of R can be less sensitive to this inhibition. (B) Model of activation of R proteins from OFF state to the ON state through a transition (T) state. High temperature might suppress the change from the T state to the ON state which involves nucleotide binding. Mutations like *snc1-1* and *snc1-3* could promote different step of progression from the OFF to the ON state. Mutations like *snc1-5* might compromise the change from the T state to the ON state.

We propose that temperature sensitivity in defense responses is largely mediated through the NB-LRR coding genes. Plant immunity is triggered when the total R or R-like activity, a multiplication of its amount and its protein activity, is above a threshold (Fig. 7A). For the R or R-like protein, its activity is intrinsically temperature sensitive. Consequently the total R activity would be below the threshold at elevated temperature, resulting in no defense. In contrast, the mutant R or R like proteins like SNC1-3 have reduced temperature sensitivity and therefore could induce heat-stable defense responses. The temperature sensitivity could be intrinsic to the protein itself or could be mediated by R-interacting chaperons whose homeostasis is affected by temperature. An elevated temperature might also reduce the R amount and thus total R activity [21],[28]. An apparent reduction of SNC1 wild-type proteins at elevated temperature is observed in transient Nb expression system and transgenic Arabidopsis (Fig. 4C, S2). To what extent this reduction of protein level contributes to the reduced defense responses at elevated temperature is yet to be determined.

It is not well understood how the heat-stable mutations such as *snc1-3* affect activities of NB-LRR proteins and how *snc1-5* reverts the heat-stable activities. Although the *snc1-3* mutation appears to induce autoactivation as suggested by the *SNC1-3* transgenic plants (Fig. 2), the Y646K and N648D mutations in N did not induce cell death in the absence of elicitors and therefore are probably not constitutively autoactive. The E640K (*snc1-3*) mutation in SNC1 and Y646K in N probably do not induce a local post-translational protein modification because the N648D mutation in the N protein cause heat-stable activity as well as Y646K. These mutations do not appear to generate a local nuclear localization signal as shorter versions of the mutant SNC1:GFP proteins did not confer nuclear localization.

It has been hypothesized that activation of the NB-LRR R proteins opens the NB domain and possibly allows interaction of the amino-terminal domain with downstream signaling molecules [31],[32]. We hypothesize that R proteins assume at least one transitional conformation (named T) between the OFF state to the ON state (Fig. 7B). Glycine close to the GLPL motif is required for the change from T state to the ON state and high temperature inhibits the change from the T state to ON state. The elicitors and mutations like *snc1-1* promotes the change from the OFF state to state T, while mutations like *snc1-3* might enhance the transition from state T to ON.

Nuclear localization is probably an immediate subsequent event after the R protein assumes the ON form. Regulated nucleo-cytoplasmic partitioning of key components is essential in hormone signaling, light signaling, temperature signaling, and plant-pathogen interactions [33],[34],[35],[36]. Temperature has been shown to influence the localization of regulators of temperature signaling. For instances, HSFA1, a heat shock transcription factor, is equally distributed between cytoplasm and nucleus at a normal growth temperature and is predominantly nuclear after a heat shock [37]. HOS1, a negative regulator of cold response, is cytoplasmic at a normal temperature but is nuclear after an exposure to a low temperature [38]. How and to what extent temperature influences nuclear localization of proteins will be an interesting subject to explore further.

Other mechanisms for temperature sensitivity must exist in plant immunity. For instance, basal resistance is inhibited by a higher growth temperature [6], indicating regulatory components other than *R* genes are modulated by temperature as well. That the NB-LRR R proteins can mediate the temperature sensitivity in disease resistance suggests that plants utilize different temperature sensors for different growth, development, and stress response processes. Future studies should further reveal the molecular basis of temperature sensitivity of *R* genes and more generally, of temperature modulation of gene expression, protein activities, and protein localization. The current climate change is causing increased range and severity of plant diseases [39]. The knowledge gained from this study will potentially provide us with tools to engineer crop plants with heat-stable disease resistance and with better adaptation to climate change.

## Materials and Methods

### Plant material and growth condition

The *Arabidopsis thaliana* plants were grown in soil at 22°C or 28°C under constant light (approximately 100 µmol m^−2^ sec^−1^) with a relative humidity between 40% and 60% for morphological phenotypic and gene expression analysis. Plants used for pathogen tests are grown under a photoperiod of 12 hr light/12 hr dark. Arabidopsis seedlings used for protoplast transformation were grown on solid medium with 1/2 MS salts, 2% sucrose, and 0.8% agar and under a photoperiod of 8 hr light/16 hr dark. *Nicotiana tobaccum* (Nt) and *Nicotiana benthamiana* (Nb) plants were grown in the greenhouse for three to four weeks before they were acclimated to 22°C on lab bench for at least a day before being used for cell death assay.

### Mutant screen and map-based cloning

The *snc1-1* seeds were treated with 0.25% EMS (ethane methyl sulfonate) for 12 hours. Approximately 40,000 M2 plants (derived from 4,000 M1) were screened at 28°C for *int* mutants with the 22°C *snc1-1* like dwarf phenotype.

The *snc1-4* seeds were mutagenized similarly. The M2 plants were screened for *rit* (*revertant of int*, i.e. wild-type-looking) mutants at 28°C.

The F2 populations for mapping the *int* or *rit* mutations were derived from genetic crossing between the mutants in the Col-0 background to wild-type plants in the Ws-2 background. Bulked segregation analysis was performed on pools of 40 plants with SSLP, CAPS, and dCAPS markers between Col-0 and Ws-2 [40].

### Generation of constructs

For the *pSNC1::SNC1:GFP* construct, a StuI site was added to the genomic fragment of *SNC1* before the stop codon via polymerase chain reaction (PCR). An EcoRI and StuI digested fragment of this product was ligated in frame with GFP to generate a 3′SNC1:GFP construct. The PstI and EcoRV digested fragment of the 5′ region of SNC1 from the BAC clone F5D3 (from Arabidopsis Biological Resource Center) and the EcoRV and PstI digested fragment of the 3′SNC1:GFP construct were ligated to the PstI site of pCAMBIA1300 to generate the *pSNC1::SNC1:GFP* construct. The SNC1-1, SNC1-3, and SNC1-4 mutations were introduced into *pSNC1::SNC1:GFP* through site-directed mutagenesis with the ‘QuikChange’ kit according to manufacture's instruction (Stratagene).

For the SNC1:GFP constructs, a 5.5 kb fragment containing the coding region and the 3′UTR of *SNC1* was isolated from the BAC clone F5D3. A BamHI restriction site was added to this genomic fragment before the stop codon of *SNC1*. The NcoI and BamHI digested fragment containing the *SNC1* coding region without the stop codon was inserted into the pSAT-N1 vector [41] to generate the *p35S::SNC1:GFP* construct. The SNC1-1, SNC1-3, SNC1-4, and SNC1-5 mutations were introduced into *p35S::SNC1:GFP* through site-directed mutagenesis as described above. The cassettes of various *p35S::SNC1:GFP* were excised at the PI-PspI restriction sites and cloned into the pHPT binary vector [41] to generate *pHPT-SNC1:GFP*, *pHPT-SNC1-1:GFP*, *pHPT-SNC1-3:GFP*, and *pHPT-SNC1-4:GFP* constructs.

For the SNC1:NES:GFP constructs, two complementary oligonucleotides encoding nuclear export signal (NES) LALKLAGLDI were synthesized with a BamHI restriction site added to both ends of the primers. NES-F: 5′GATCCTGGCTTTGAAGTTAGCTGGTTTGGATATCAA 3′. NES-R: 5′ GATCTTGATATCCAAACCAGCTAACTTCAAAGCCAG 3′. Synthetic oligo-nucleotides were denatured, annealed, and inserted into the BamHI restriction site between SNC1 and GFP to generate the *p35S::SNC1-3:NES:GFP* and *p35S::SNC1-4:NES:GFP*. The two *p35S::SNC1:NES:GFP* cassettes were cloned into the pHPT binary vector to generate *pHPT-SNC1-3:NES:GFP*, and *pHPT-SNC1:NES:GFP* constructs as described above.

The wild-type *N* gene for mutagenesis was described previously [26]. It is a HA tagged genomic fragment of the *N* gene under the control of a 35S promoter. This *N* gene was subject to site-directed mutagenesis with the ‘QuikChange’ kit as described above.

All primers sequences will be available upon request.

### Transgenic plants generation

*Agrobacterium tumefaciens* stains of GV3101 (Koncz and Schell, 1986) carrying various *SNC1* constructs were used to transform wild-type Col-0 plants via standard floral dipping method [42]. Primary transformants were selected on solid medium containing hygromycin.

### Protoplast transformation

Protoplast isolation and transformation were carried out as previously described [43]. In brief, protoplasts were generated from wild-type and mutant seedlings grown on plates. After transformation, protoplasts were incubated at specific temperatures and the GFP signals were observed from 12 hours to 48 hours.

### Transient expression in Nb and Nt

The binary vectors were transformed into *Agrobacterium tumefaciens* strain C58C1 [20] for transient expression. Agrobacterial cultures were grown overnight to OD_600_ of 1.0 in liquid LB. Cells were then collected by centrifugation and resuspended in the induction medium ( 10 mM MES, pH 5.7, 10 mM MgCl_2_, 200 µM acetosyringone) to OD_600_ of 0.8. After incubating at room temperature for 3 hrs, the Agrobacterial cells were infiltrated into the abaxial surface of Nb or Nt leaves using 1 ml needleless syringes. On average, four spots were used to infiltrate one whole Nb leaf. Infiltrated plants were subsequently incubated at 22°C or 28°C before the infiltrated leaves were examined for GFP signals under a microscope (Zeiss AXIO 2 plus or Leica TCS SP5) within a 48 hr period after inoculation.

### RNA analysis

Total RNAs were extracted using Tri Reagent (Molecular Research, Cincinnati, OH) from leaves of 3-week-old plants. Twenty micrograms of total RNAs per sample were used for RNA gel blot analysis according to standard procedure [44].

### Pathogen resistance assay

*P. syringae pv. tomato* DC3000 was grown 2 to 3 days on the KB medium and resuspended at 10^5^ cfu (colony forming unit) per ml in a solution of 10mM MgCl_2_ and 0.02% Silwet L-77. Two- week-old seedlings were dip inoculated with bacteria and kept covered for 1 h. The amount of bacteria in plants was analyzed at 1 h after dipping (day 0) and 3 days after dipping (day 3). Bacterial growth was determined as described previously [45].

## Supporting Information

Figure S1 Amino acid sequences of the SNC1 proteins. The TIR, NB-ARC, and LRR domains are colored. Mutated residues of *snc1-1, snc1-3*, and *snc1-5* are underlined.
(0.02 MB DOC)

Figure S2 Expression levels of the SNC1 proteins do not correlate with their protein activity at high temperature. Shown is Western blot analysis of SNC1:GFP expression in Arabidopsis transgenic plants with *pSNC1::SNC1:GFP* constructs by anti-GFP antibody. Lines with a 28°C rescued phenotype are indicated by red color and those with a non-rescued phenotype are indicated by black color. A cross-hybridization band was used as loading control. Abbreviations: pWT: *pSNC1::SNC1:GFP*; p-1: *pSNC1::SNC1-1:GFP*; p-3: *pSNC1::SNC1-3:GFP*; p-4: *pSNC1::SNC1-4:GFP.*
(0.06 MB PDF)

Text S1 Supporting materials and methods
(0.03 MB PDF)

## References

[1] Long SP, Woodward FI (1988). Plants and temperature; Long SP, Woodward FI, editors..

[2] Penfield S (2008). Temperature perception and signal transduction in plants.. New Phytol.

[3] Garrett KA, Dendy SP, Frank EE, Rouse MN, Travers SE (2006). Climate change effects on plant disease: genomes to ecosystems.. Annu Rev Phytopathol.

[4] Dropkin V (1969). The necrotic reaction of tomatoes and other hosts resistant to Meloidogyne: reversal by temperature.. Phytopathology.

[5] Szittya G, Silhavy D, Molnar A, Havelda Z, Lovas A (2003). Low temperature inhibits RNA silencing-mediated defence by the control of siRNA generation.. Embo J.

[6] Wang Y, Bao Z, Zhu Y, Hua J (2009). Analysis of temperature modulation of plant defense against biotrophic microbes.. Mol Plant Microbe Interact.

[7] Whitham S, McCormick S, Baker B (1996). The N gene of tobacco confers resistance to tobacco mosaic virus in transgenic tomato.. Proc Natl Acad Sci U S A.

[8] Hwang CF, Bhakta AV, Truesdell GM, Pudlo WM, Williamson VM (2000). Evidence for a role of the N terminus and leucine-rich repeat region of the Mi gene product in regulation of localized cell death.. Plant Cell.

[9] Xiao S, Brown S, Patrick E, Brearley C, Turner JG (2003). Enhanced transcription of the *Arabidopsis* disease resistance genes *RPW8.1* and *RPW8.2* via a salicylic acid-dependent amplification circuit is required for hypersensitive cell death.. Plant Cell.

[10] de Jong CF, Takken FL, Cai X, de Wit PJ, Joosten MH (2002). Attenuation of Cf-mediated defense responses at elevated temperatures correlates with a decrease in elicitor-binding sites.. Mol Plant Microbe Interact.

[11] Yang S, Hua J (2004). A haplotype-specific Resistance gene regulated by *BONZAI1* mediates temperature-dependent growth control in Arabidopsis.. Plant Cell.

[12] Zhang Y, Goritschnig S, Dong X, Li X (2003). A gain-of-function mutation in a plant disease resistance gene leads to constitutive activation of downstream signal transduction pathways in *suppressor of npr1-1, constitutive 1*.. Plant Cell.

[13] Bomblies K, Lempe J, Epple P, Warthmann N, Lanz C (2007). Autoimmune response as a mechanism for a Dobzhansky-Muller-type incompatibility syndrome in plants.. PLoS Biol.

[14] Larkindale J, Hall JD, Knight MR, Vierling E (2005). Heat stress phenotypes of Arabidopsis mutants implicate multiple signaling pathways in the acquisition of thermotolerance.. Plant Physiol.

[15] Clarke SM, Mur LA, Wood JE, Scott IM (2004). Salicylic acid dependent signaling promotes basal thermotolerance but is not essential for acquired thermotolerance in Arabidopsis thaliana.. Plant J.

[16] Uknes S, Mauch-Mani B, Moyer M, Potter S, Williams S (1992). Acquired resistance in Arabidopsis.. Plant Cell.

[17] Lawton K, Weymann K, Friedrich L, Vernooij B, Uknes S (1995). Systemic acquired resistance in Arabidopsis requires salicylic acid but not ethylene.. Mol Plant Microbe Interact.

[18] Jirage D, Tootle TL, Reuber TL, Frost LN, Feys BJ (1999). Arabidopsis thaliana PAD4 encodes a lipase-like gene that is important for salicylic acid signaling.. Proc Natl Acad Sci U S A.

[19] Li Y, Yang S, Yang H, Hua J (2007). The TIR-NB-LRR gene SNC1 is regulated at the transcript level by multiple factors.. Mol Plant Microbe Interact.

[20] Rairdan GJ, Moffett P (2006). Distinct domains in the ARC region of the potato resistance protein Rx mediate LRR binding and inhibition of activation.. Plant Cell.

[21] Bieri S, Mauch S, Shen QH, Peart J, Devoto A (2004). RAR1 positively controls steady state levels of barley MLA resistance proteins and enables sufficient MLA6 accumulation for effective resistance.. Plant Cell.

[22] Wen W, Meinkoth JL, Tsien RY, Taylor SS (1995). Identification of a signal for rapid export of proteins from the nucleus.. Cell.

[23] Zhang Y, Li X (2005). A putative nucleoporin 96 Is required for both basal defense and constitutive resistance responses mediated by suppressor of npr1-1,constitutive 1.. Plant Cell.

[24] Palma K, Zhang Y, Li X (2005). An importin alpha homolog, MOS6, plays an important role in plant innate immunity.. Curr Biol.

[25] Whitham S, Dinesh-Kumar SP, Choi D, Hehl R, Corr C (1994). The product of the tobacco mosaic virus resistance gene N: similarity to toll and the interleukin-1 receptor.. Cell.

[26] Mestre P, Baulcombe DC (2006). Elicitor-mediated oligomerization of the tobacco N disease resistance protein.. Plant Cell.

[27] Burch-Smith TM, Schiff M, Caplan JL, Tsao J, Czymmek K (2007). A Novel Role for the TIR Domain in Association with Pathogen-Derived Elicitors.. PLoS Biol.

[28] Yang M, Wardzala E, Johal GS, Gray J (2004). The wound-inducible Lls1 gene from maize is an orthologue of the Arabidopsis Acd1 gene, and the LLS1 protein is present in non-photosynthetic tissues.. Plant Mol Biol.

[29] Li Y, Pennington BO, Hua J (2009). Multiple R-like genes are negatively regulated by BON1 and BON3 in arabidopsis.. Mol Plant Microbe Interact.

[30] Jablonska B, Ammiraju JS, Bhattarai KK, Mantelin S, Martinez de Ilarduya O (2007). The Mi-9 gene from Solanum arcanum conferring heat-stable resistance to root-knot nematodes is a homolog of Mi-1.. Plant Physiol.

[31] Rathjen JP, Moffett P (2003). Early signal transduction events in specific plant disease resistance.. Curr Opin Plant Biol.

[32] Takken FL, Albrecht M, Tameling WI (2006). Resistance proteins: molecular switches of plant defence.. Curr Opin Plant Biol.

[33] Merkle T (2003). Nucleo-cytoplasmic partitioning of proteins in plants: implications for the regulation of environmental and developmental signalling.. Curr Genet.

[34] Meier I (2005). Nucleocytoplasmic trafficking in plant cells.. Int Rev Cytol.

[35] Xu XM, Meier I (2008). The nuclear pore comes to the fore.. Trends Plant Sci.

[36] Cheng YT, Germain H, Wiermer M, Bi D, Xu F (2009). Nuclear Pore Complex Component MOS7/Nup88 Is Required for Innate Immunity and Nuclear Accumulation of Defense Regulators in Arabidopsis.. Plant Cell.

[37] Scharf KD, Heider H, Hohfeld I, Lyck R, Schmidt E (1998). The tomato Hsf system: HsfA2 needs interaction with HsfA1 for efficient nuclear import and may be localized in cytoplasmic heat stress granules.. Mol Cell Biol.

[38] Ishitani M, Xiong L, Lee H, Stevenson B, Zhu JK (1998). HOS1, a genetic locus involved in cold-responsive gene expression in arabidopsis.. Plant Cell.

[39] Evans N, Baierl A, Semenov MA, Gladders P, Fitt BD (2008). Range and severity of a plant disease increased by global warming.. J R Soc Interface.

[40] Lukowitz W, Gillmor CS, Scheible WR (2000). Positional cloning in Arabidopsis. Why it feels good to have a genome initiative working for you.. Plant Physiol.

[41] Tzfira T, Tian GW, Lacroix B, Vyas S, Li J (2005). pSAT vectors: a modular series of plasmids for autofluorescent protein tagging and expression of multiple genes in plants.. Plant Mol Biol.

[42] Clough SJ, Bent AF (1998). Floral dip: a simplified method for Agrobacterium-mediated transformation of *Arabidopsis thaliana*.. Plant J.

[43] Zhai Z, Sooksa-nguan T, Vatamaniuk OK (2009). Establishing RNA interference as a reverse-genetic approach for gene functional analysis in protoplasts.. Plant Physiol.

[44] Sambrook J, Fritsch EF, Maniatis T (1989). Molecular Cloning, a Laboratory Manual..

[45] Yang H, Yang S, Li Y, Hua J (2007). The Arabidopsis BAP1 and BAP2 genes are general inhibitors of programmed cell death.. Plant Physiol.

