# Supplementary material for: Temperature Modulates Plant Defense Responses through NB-LRR Proteins
Source: PLoS Pathog. 2010 Apr 1;6(4):e1000844. doi: 10.1371/journal.ppat.1000844 (PMC2848567; doi:10.1371/journal.ppat.1000844)
Supplement: Figure S1 — Amino acid sequences of the SNC1 proteins. The TIR, NB-ARC, and LRR domains are colored. Mutated residues of snc1-1, snc1-3, and snc1-5 are underlined. (0.02 MB DOC) [file ppat.1000844.s001.doc]

**MEIASSSGSRRYDVFPSFRGEDVRDSFLSHLLKELRGKAITFIDDEIERSRSIGPELLSAIKESRIAIVI**

**FSKNYASSTWCLNELVEIHKCYTNLNQMVIPIFFHVDASEVKKQTGEFGKVFEETCKAKSEDEKQSWKQA**

**LAAVAVMAGYDLRKWPSEAAMIEELAEDVLRKTMTPSDDFGDLVGIENHIEAIKSVLCLESKEARIMVGI**

**WGQSGIGKSTIGRALYSKLSIQFHHRAFITYKSTSGSDVSGMKLRWEKELLSEILGQKDIKIEHFGVVEQ**

**RLKQQKVLILLDDVDSLEFLKTLVGKAEWFGSGSRIIVITQDRQLLKAHEIDLIYEVEFPSEHLALTMLC**

**RSAFGKDSPPDDFKELAFEVAKLAGNLPLGLSVLGSSLKGRTKEWWMEMMPRLRNGLNGDIMKTLRVSYD**

**RLHQKDQDMFLYIACLFNGFEVSYVKDLLKDNVGFTMLTEKSLIRITPDGYIEMHNLLEKLGREIDRAKS**

**KGNPGKRRFLTNFEDIHEVVTEKTGTETLLGIRLPFEEYFSTRPLLIDKESFKGMRNLQYLEIGYYGDLP**

**QSLVYLPLKLRLLDWDDCPLKSLPSTFKAEYLVNLIMKYSKLEKLWEGTLPLGSLKEMNLRYSNNLKEIP**

**DLSLAINLEELDLVGCKSLVTLPSSIQNATKLIYLDMSDCKKLESFPTDLNLESLEYLNLTGCPNLRNFP**

**AIKMGCSDVDFPEGRNEIVVEDCFWNKNLPAGLDYLDCLTRCMPCEFRPEQLAFLNVRGYKHEKLWEGIQ**

**SLGSLEGMDLSESENLTEIPDLSKATKLESLILNNCKSLVTLPSTIGNLHRLVRLEMKECTGLEVLPTDV**

**NLSSLETLDLSGCSSLRSFPLISTNIVWLYLENTAIEEIPSTIGNLHRLVRLEMKKCTGLEVLPTDVNLS**

**SLETLDLSGCSSLRSFPLISESIKWLYLENTAIEEIPDLSKATNLKNLKLNNCKSLVTLPTTIGNLQKLV**

**SFEMKECTGLEVLPIDVNLSSLMILDLSGCSSLRTFPLISTNIVWLYLENTAIEEIPSTIGNLHRLVKLE**

**MKECTGLEVLPTDVNLSSLMILDLSGCSSLRTFPLISTRIECLYLQNTAIEEVPCCIEDFTRLTVLMMYC**

**CQRLKTISPNIFRLTRLELADFTDCRGVIKALSDATVVATMEDHVSCVPLSENIEYIWDKLYHLPSKLNF**

**NDVEFKFCCSNRIKECGVRLMYVSQEENNQQTTRSEKRMRMTSGTSEEDINLPYGLIVADTGLAALNMEL**

**SLGQGEPSSSTSLEGEALCVDYMITEEQDKGIPILFPVSGN**

**Color code: TIR NB-ARC LRR**
