# Supplementary figures and images for: Temperature Modulates Plant Defense Responses through NB-LRR Proteins
Source: PLoS Pathog. 2010 Apr 1;6(4):e1000844. doi: 10.1371/journal.ppat.1000844 (PMC2848567; doi:10.1371/journal.ppat.1000844)

Supplemental Figure 2

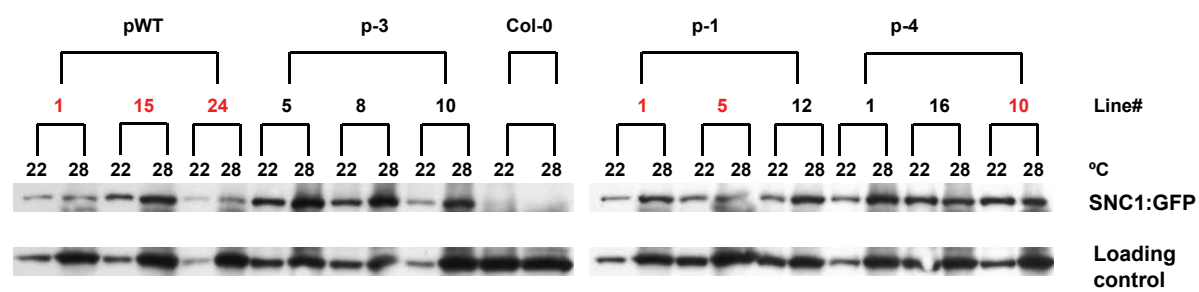

Supplement: Figure S2 — Expression levels of the SNC1 proteins do not correlate with their protein activity at high temperature. Shown is Western blot analysis of SNC1:GFP expression in Arabidopsis transgenic plants with pSNC1::SNC1:GFP constructs by anti-GFP antibody. Lines with a 28°C rescued phenotype are indicated by red color and those with a non-rescued phenotype are indicated by black color. A cross-hybridization band was used as loading control. Abbreviations: pWT: pSNC1::SNC1:GFP; p-1: pSNC1::SNC1-1:GFP; p-3: pSNC1::SNC1-3:GFP; p-4: pSNC1::SNC1-4:GFP. (0.06 MB PDF) [file ppat.1000844.s002.pdf]
