# Supplementary material for: Temperature Modulates Plant Defense Responses through NB-LRR Proteins
Source: PLoS Pathog. 2010 Apr 1;6(4):e1000844. doi: 10.1371/journal.ppat.1000844 (PMC2848567; doi:10.1371/journal.ppat.1000844)
Supplement: Text S1 — Supporting materials and methods (0.03 MB PDF) [file ppat.1000844.s003.pdf]

Protein analysis

Leaf tissues were ground in liquid nitrogen and extracted with a buffer containing 50 mM Tris-HCl pH7.5, 150 mM NaCl, 1 mM EDTA, 10% Glycerol, 1% Triton X-100, 5 mM DTT, and protease inhibitors (1 mM PMSF, 2 μg/ml aprotinin, 10 μg/ml leupeptin, and 1 μg/ml pepstatin). Total protein extracts were boiled with 4 X sample buffer (125 mM Tris-HCl pH6.8, 10% SDS, 50% glycerol, 1 mM DTT and 0.4% Bromophenol blue) for 10 minutes and spun at 12,000 rpm in a microcentrifuge for 5 minutes. The supernatants were separated on 8% SDS-PAGE gels. Western blot analysis was carried out following standard procedures with an anti-GFP monoclonal antibody (Covance, CA).
